# Molecular Epidemiology Questions Transmission Pathways Identified During the Year 2000 Outbreak of Classical Swine Fever in the UK

**DOI:** 10.3389/fmicb.2022.909396

**Published:** 2022-06-28

**Authors:** Rebecca Strong, Stephen McCleary, Sylvia Grierson, Bhudipa Choudhury, Falko Steinbach, Helen R. Crooke

**Affiliations:** Virology Department, Animal and Plant Health Agency, APHA-Weybridge, Addlestone, United Kingdom

**Keywords:** classical swine fever virus, pigs, full genome sequencing, molecular epidemiology, outbreak transmission pathways

## Abstract

The last outbreak of classical swine fever (CSF) in the UK occurred in 2000. A total of 16 domestic pig holdings in the East Anglia region were confirmed as infected over a 3-month period. Obtaining viral genome sequences has since become easier and more cost-effective and has accordingly been applied to trace viral transmission events for a variety of viruses. The rate of genetic evolution varies for different viruses and is influenced by different transmission events, which will vary according to the epidemiology of an outbreak. To examine if genetic changes over the course of any future CSF outbreak would occur to supplement epidemiological investigations and help to track virus movements, the E2 gene and full genome of the virus present in archived tonsil samples from 14 of these infected premises were sequenced. Insufficient changes occurred in the full E2 gene to discriminate between the viruses from the different premises. In contrast, between 5 and 14 nucleotide changes were detected between the genome sequence of the virus from the presumed index case and the sequences from the other 13 infected premises. Phylogenetic analysis of these full CSFV genome sequences identified clusters of closely related viruses that allowed to corroborate some of the transmission pathways inferred by epidemiological investigations at the time. However, other sequences were more distinct and raised questions about the virus transmission routes previously implicated. We are thus confident that in future outbreaks, real-time monitoring of the outbreak *via* full genome sequencing will be beneficial.

## Introduction

Classical swine fever (CSF) is a contagious febrile disease of pigs that remains a severe socio-economic threat to the global pig industry. The virus has been eradicated from many developed countries, but it continues to cause economic losses in endemic regions. The reintroduction of the virus into disease-free areas has a major impact, as highlighted by the re-emergence of the disease in Japan in 2018 after 26 years of freedom (Ganges et al., 2020; Shimizu et al., 2021). The virus is transmitted directly between pigs, either by vertical or horizontal transmission, including by contact with wild boar, or indirectly by contaminated food, objects, or people. Timely interventions can rapidly identify infected premises, reduce the spread and length of an outbreak, provide a significant economic and welfare benefit, and facilitate the earlier restoration of trade in pig meat and products (Postel et al., 2018).

**Figure 1:**
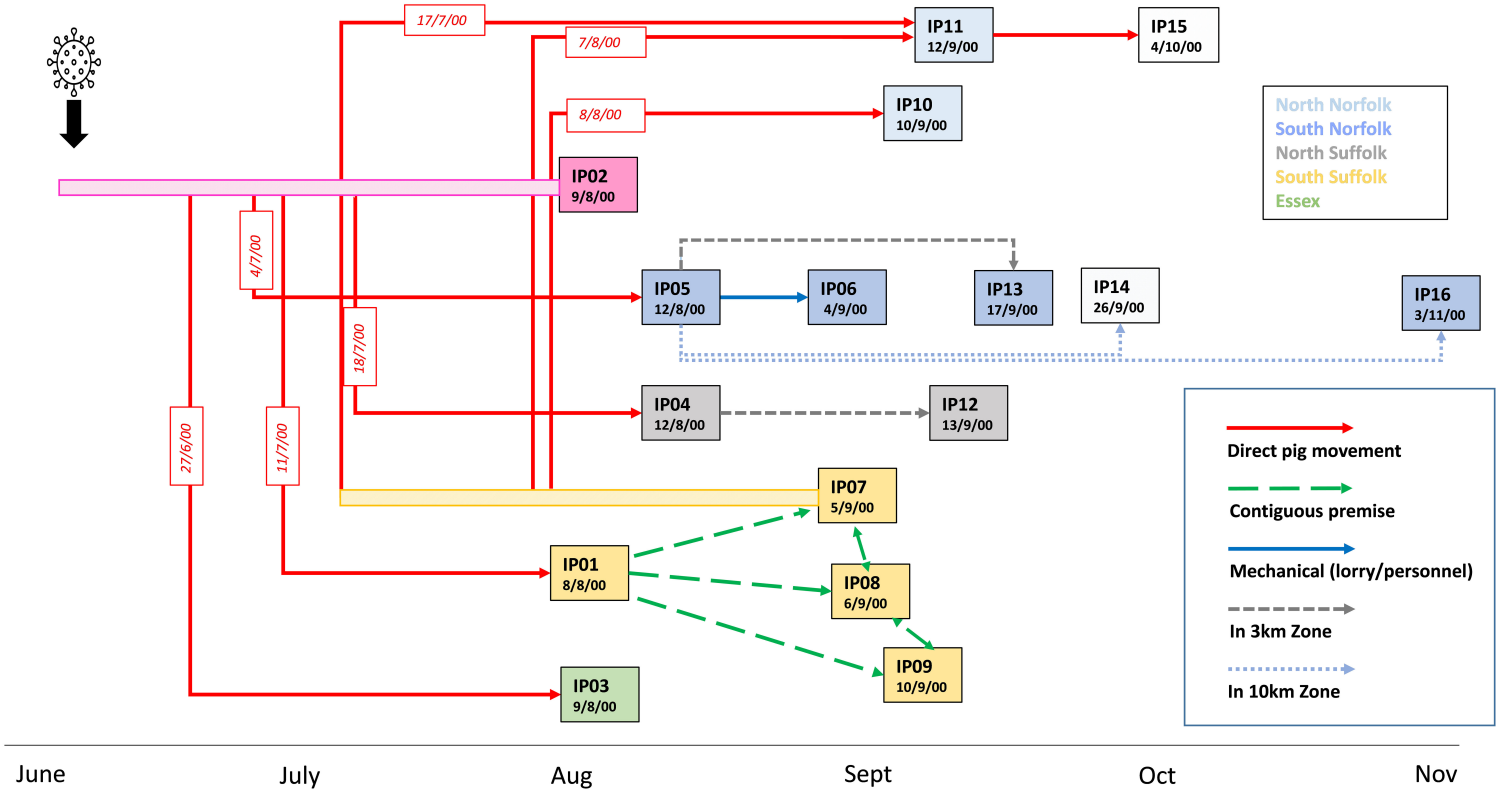
Epidemiological links and timeline of the outbreak of CSF in the UK in 2000. Dates indicate the time of confirmation of CSF disease on the 16 premises (IP01-IP16) detected as infected during the 2000 outbreak. Premises were assigned IP numbers in the order of disease confirmation. Rectangle colors indicate the location of the premises within 5 geographical areas of the East Anglia region. Premises for which samples were not available are indicated by non-coloured rectangles. The most likely routes of transmission identified by epidemiological investigations are indicated by arrows. Dates of known direct movements of animals are indicated in red boxes. Bars before IP02 and IP07 indicate a potentially infectious period before disease confirmation on these premises from which direct animal movements occurred.

The last outbreak of CSF in the UK occurred in 2000 in the East Anglia region, a high pig dense area that was not known to have a feral pig population. A total of 264 premises were investigated for CSF suspicion although only 16 premises (designated IP01 to IP16) were confirmed as infected over a 3-month period. The outbreak was controlled by culling of infected and contact premises and vaccination was not applied. The first case (IP01) was confirmed on 8 August 2000. However, substantial illness, attributed to porcine circovirus (PCV-2)-associated disease, had been present since 11 July when weaner animals were introduced from an outdoor breeder unit (IP02) which was, at the time, accordingly thought to have been the origin of the outbreak (Figure 1). A routine veterinary visit of IP02 on 19^th^ June did not find evidence of clinical disease. Signs noted were attributed to heat stress, and production records and mortality rates did not give cause for concern. However, it is thought that the disease was present on this premise since at least from early June (Mackinnon, 2001). Records indicated that the first signs of illness could have been found in a sow which was recorded as being unwell on 20 June and died on 25 July. Weaners from IP02 were sold to a large pig producing business that owned numerous farms, including IP01, IP03, IP04, and IP05. A batch of weaners was transferred from IP02 on 27 June to a rearing unit in Essex (IP03). IP04 and IP05, located in Suffolk and Norfolk, received animals from IP02 on 18 July and 4 July, respectively (Paton, 2002).

In early September, a cluster of further cases was detected in the south of Suffolk, close to IP01. IP07, an outdoor breeding herd with land adjacent to IP01, was confirmed as infected on 5 September. IP08, which had 5,000 growers and finishers, lies to the north of IP07 and had outdoor growers housed on land contiguous to IP07. IP09 also had outdoor sows, gilts, and boars located close to IP01. It was concluded that lateral local spread infected these premises, possibly due to the scavenging of dead pigs from IP01 by wild animals before CSF was confirmed (Mackinnon, 2001; Paton, 2002). A feature leading to delays in the detection of disease, especially in outdoor breeding units, was the insidious nature of clinical signs induced by the moderately virulent strain in adult pigs.

Two distantly located premises, IP10 and IP11, were confirmed on the 10^th^ and 11^th^ of September. Both these north Norfolk premises had received pigs from IP07; IP10 received weaner pigs on 8 August, whereas IP11 received breeding animals in both July and August. Pigs were sent from IP11 to IP15, which was later diagnosed on serological evidence. There was less information on potential transmission routes into the remaining infected premises; IP06, IP13, IP14, and IP16 were all located within 10 km of IP5, and IP12 is located close to IP04, suggesting that local spread may have occurred.

Genetic analysis of virus genomes can help us to understand disease transmission pathways to inform effective disease control policy decisions. However, for genetic data to be useful for molecular epidemiology, sufficient genetic diversity needs to occur over a relevant epidemiological timescale. Traditionally, parts of the 5'NTR, E2, and sometimes the NS5B encoding regions of the CSFV genome, are sequenced to determine the CSFV genotype (Paton et al., 2000). These target regions are easily obtained and provide rapid information on the potential source of a newly introduced virus. However, the short sequence length does not allow discrimination between closely related viruses and is of limited use to trace virus movements within an outbreak. This lead Postel and colleagues to propose an improved phylogenetic strategy, based on the analysis of the full-length E2 sequences (Postel et al., 2012). In the past decade, sequencing technologies have advanced such that obtaining full-length sequence of viral genomes is equally cost-efficient and rapid. This enhances the potential for full genome analysis to help inform about transmission events occurring during an outbreak.

To gain an understanding of the utility of generating full genome sequence data during a CSF outbreak, we obtained sequences of the E2 gene and viral genomes from archived tissue samples. Phylogenetic analysis of full genome data supported many, but not all, of the likely transmission pathways identified by epidemiological investigations at the time. The data also reveal additional details to the transmission pathways discovered through field epidemiology at the time.

## Materials and Methods

### RNA Extraction From Tonsil

Archived tonsil samples that had been stored at −80°C were available from individual animals from premises IP01-IP05 and IP16, whereas samples from premises IP06–IP13 were stored as pools of tissues from up to 4 pigs. A pooled sample (IP01-7.2) was also available from IP01. No samples were available from IP14 and IP15. To maximize the proportion of sequence data derived from the viral genome, as opposed to contaminating host nucleic acid, tonsil samples (~3–5 mm^3^) were added to 300 μl of lysis buffer (5 mM MgCl_2_, 50 mM Trizma, in PBS) containing 5 U/μl of the endonuclease Omnicleave (Cambio). After incubation at room temperature for 30 min, the samples were homogenized in a Precellys 24 homogenizer in tubes with ceramic beads (CR28) with 3 cycles of 5,000 rpm for 45 s, with 30 s pauses between each cycle. Alternatively, the samples were homogenized using a GentleMax homogenizer and dissociated for 54 s using the manufacturer's program RNA_01.01. The homogenized samples were then clarified by centrifugation at 14,000 × *g* for 2 min, the supernatant was mixed with 1 ml of the Trizol reagent (Thermofisher), and RNA was extracted according to the manufacturer's instructions.

### Sequencing of E2 and the Full Coding Region of the CSFV Genome

The full E2 coding region was amplified from one sample from each premise, except for IP06, with Phusion polymerase (Thermofisher) with primers 2250F and 3710R (Postel et al., 2012) after preparation of cDNA with thermoscript III reverse transcriptase (Fisher scientific). The DNA sequence was obtained by Sanger sequencing using primers 3010F, 2919F, and 3000R as described (Postel et al., 2012). Almost full-length viral genome sequences were obtained by preparation of cDNA with a Superscript IV first-strand synthesis system (Thermofisher) followed by NEBNext® Ultra II non-directional RNA second-strand synthesis (NEB). To prepare a sequencing library, 1 ng of cDNA was added to the Nextera XT library kit and the samples were subsequently sequenced with an Illumina MiSeq. All paired-end sequence reads were trimmed using Trimmomatic (v0.39) (Bolger et al., 2014). Adapter sequences were removed and the headcrop option was used to remove 16 bases from the start of reads. Sliding window size and quality settings of 5:20 and minimum length trimming of 40 were applied. The CSFV genome sequence was obtained by *de novo* assembly (IVA v1.0.8 using default settings) (Hunt et al., 2015) of the sequence from IP02, the assumed index case, and checked by reference guided mapping against itself (BWA-MEM) v0.7.17) (Li and Durbin, 2009). The SF02 sequence was then used as a reference sequence for reference guided mapping of the remaining sequences. The sequences obtained from IP01–IP16 were designated SF01–SF16, respectively. The sequence from the pooled sample from IP01 (IP01-7.2) was designated SF01-7.2. Consensus sequences were called using iVar (Grubaugh et al., 2019a) with a minimum quality score of 20 and a depth of 1 and a frequency threshold of 0.6. A small region in the 3'UTR that was not obtained by Illumina sequencing for the sample from IP12 was amplified by PCR using primers 5′ GCAAGACGGGAAATAGGTAC 3′ and 5′ TTTTCCCTCCAGCTAAAGTG 3′ and the resulting product was sequenced by Sanger sequencing. The resultant sequences were submitted to DDBJ.

### Phylogenetic Analysis

Sequences were aligned using MegaX using the Clustal W algorithm. The evolutionary history was inferred using the Neighbor-Joining method. The bootstrap test was applied to infer confidence values; the percentage of trees in which the associated taxa clustered together in 1,000 replicates was expressed as a percentage, with values ≥70% displayed at the tree nodes. The evolutionary distances were computed using the Maximum Composite Likelihood method. The sequence of the genotype 2.1 Penevezys strain CSF1048, isolated in Lithuania in 2009, was used as an outgroup (Genbank accession HQ148063).

## Results

The analysis of the complete E2 gene indicated that most of the samples contained viruses with identical E2 sequences which, in turn, were also identical to the previously published sequence of the virus isolate CSF0708 obtained from IP03 (JQ441582). However, the samples from IP08 and IP12, which were confirmed positive in September, and IP16, which was the last premise detected 3 months after the first case, had viruses with one (IP12 and IP16) or two nucleotide changes (IP08) compared to the sequence from IP02. Accordingly, the use of the E2 gene would not suffice for in-depth molecular epidemiological approaches (data not shown).

Near full-length sequences were obtained for all 15 samples: 12,136 nucleotides encoding the polyprotein region and 349/102 nucleotides of the 5'/3' UTRs, respectively (DDBJ accession numbers LC702327–LC702341). The changes in the E2 gene in SF12, SF16, and SF08 identified by sequencing of PCR product were present in the Illumina generated sequences, confirming that they were not the result of a Phusion polymerase error.

The alignment of the sequences encoding the almost full-length genome confirmed that the viruses in all 15 samples were highly similar (>99.8% identical), with only 5–14 nucleotide changes compared to the sequence from IP02, which was taken as the baseline as it was considered to be the first infected premise (Supplementary Table 1). Nucleotide changes were present in all regions of the genome, except for the regions encoding the core and NS4A proteins. The changes in the sequence SF01-7.2 from the pooled sample were degenerate, indicating that animals contributing to the pool may have been carrying slightly different viruses. Ten non-synonymous changes were identified but, as these were not shared in sequences from different premises, there was no evidence that these amino acid changes imparted a competitive advantage.

**Figure 2:**
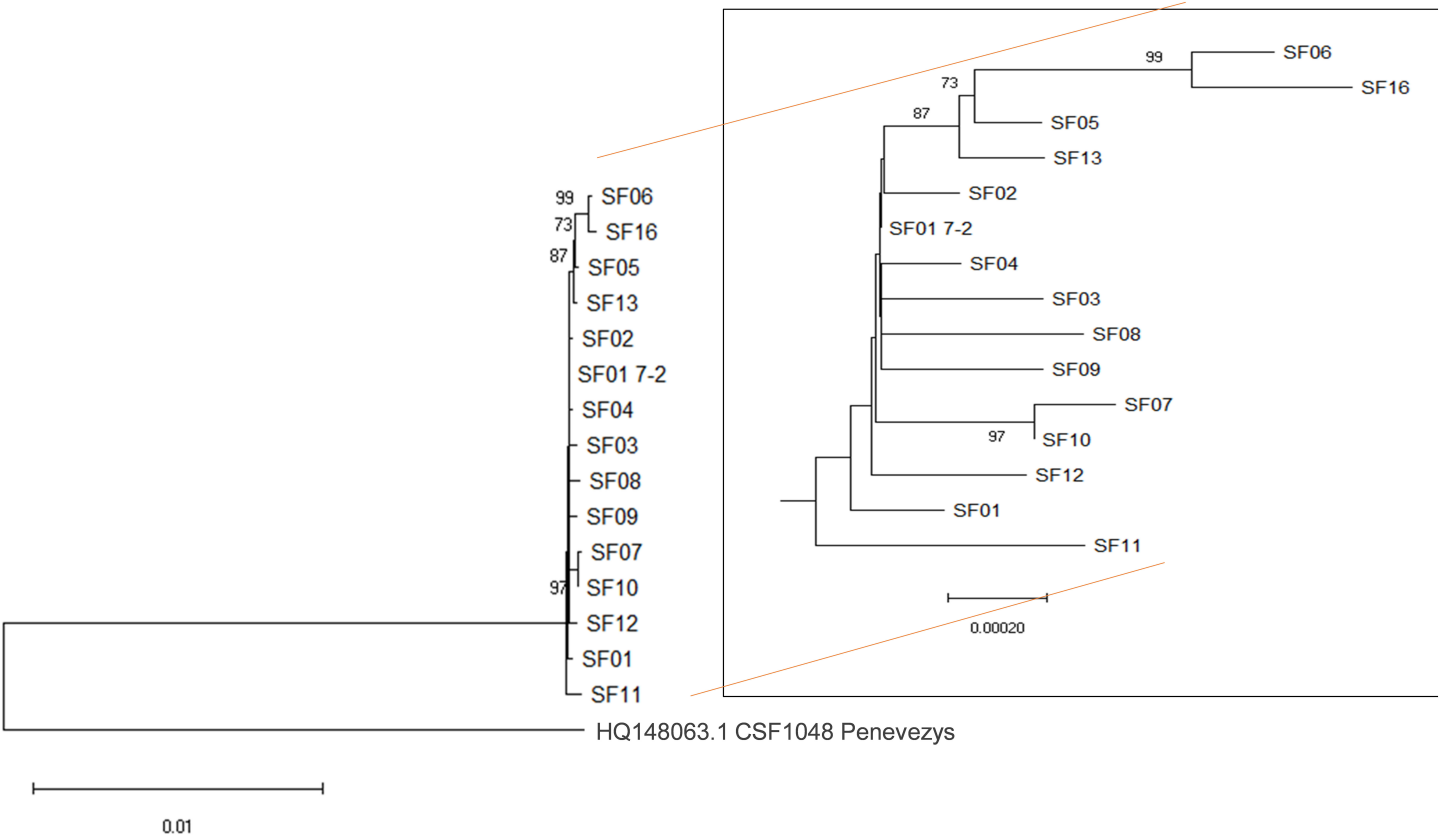
Phylogenetic analysis. Almost full-length sequences from 14 infected premises were aligned using the sequence of the closely related genotype 2.1 CSFV1048 Penevezys sequence as an outgroup. There were a total of 12,136 positions in the final dataset. The percentage of replicate trees in which the associated taxa clustered together in the bootstrap test (1,000 replicates), is shown next to the branches with values ≥ 70%. SF01 7-2 is a sequence from a pooled sample from the first premise on which CSF was confirmed, whereas SF01 is from a single animal from the same premise.

Although virus genome sequences from all infected premises were highly similar, the phylogenetic analysis revealed succinct clusters between the viruses, with sequences from IP05, IP13 IP06, and IP16 forming a separate group, and IP06 and IP16 forming a subgroup therein (Figure 2). In addition, the sequences obtained from IP07 and IP10 were distinct from the main group but were closely related to each other. Furthermore, the sequence from IP11 was distinct from all others. These phylogenetic clusters, which are supported by high bootstrap values (>80%), were not influenced by the nature of the samples (pooled vs. individual animals). In this context, it is interesting to note that there were 9 nucleotide differences between the sequences obtained from the sample from an individual animal from IP01, and the pooled sample (IP01-7.2). The SF01 sequence from the individual animal sits slightly outside the main sample group formed by sequences SF01-04, 08, and 09 but is not sufficient to form a bootstrap-supported group. It is, perhaps, not surprising that more changes are apparent in the sequence from an individual animal as changes in only one animal may be masked within the consensus sequence of a pooled sample.

**Figure 3:**
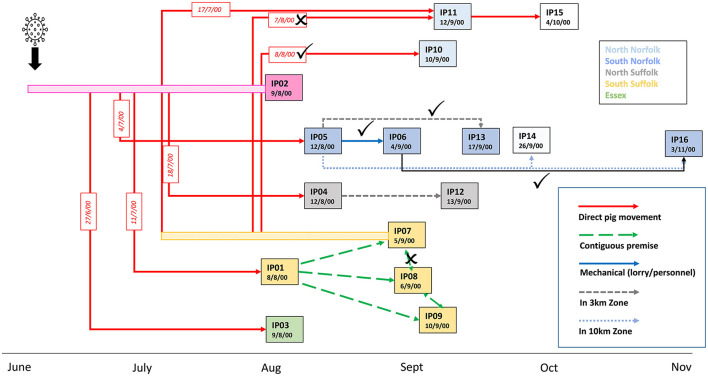
Updated model of the linkages between the affected pig holdings in the UK CSF outbreak in 2000. Transmission links identified by epidemiological investigations that are corroborated by phylogenetic analysis are indicated with ticks and links that are not supported by crosses. A new transmission route implicated by the phylogenetic analysis is indicated by the black arrow.

Phylogenetic analysis of the sequences (SF01-SF16) obtained from the different infected premises highlighted clusters of related sequences that were supportive of virus transmission routes suggested by epidemiological investigations (Figure 3). The sequences obtained from IP05, IP06, IP13, and IP16, all located in the south Norfolk area, support the hypothesis that virus spread by local transmission within the 10-km zone surrounding IP05. The sequence data suggest that IP06 was the most likely source of virus infecting IP16, which was the last premise detected, although IP14, for which samples were not available, may also have been involved in this transmission chain. The close relatedness of viruses found in IP07 and IP10 also strongly implicates the transfer of weaner pigs from IP07 to IP10 as being the source of virus introduction to IP10.

In contrast, although a direct transfer of animals between IP07 and IP11 occurred on 7 August, the sequence data indicate that the viruses on the two premises are diverse and an alternative route of spread to IP11 appears more likely. For example, the virus may have been transmitted earlier *via* the transport of breeding sows on 17 July from IP07, which would also explain the higher degree of virus evolution and outside clustering of sequence SF11, or transmission could have occurred *via* another unidentified contact with any of the earlier infected premises. Sequence information was less informative for clarifying virus transmission routes within the south Suffolk cluster of farms, IP01, IP07, IP08, and IP09, although the molecular analysis is not inconsistent with the theory of lateral spread by wildlife scavenging on carcasses left on IP01 prior to disease confirmation (Mackinnon, 2001). With SF07 being part of a genetic subgroup, the idea that there was a transmission from IP07 to the adjacent IP08 is not corroborated by the molecular analysis. Similarly, the sequence data from IP04 and IP12 do not strongly support the theory that IP12 was infected directly by the virus from the closely located IP04. What also cannot be resolved further by this molecular approach are transmissions between the premises IP01-04, 08, 09, and 12 and how the infection spreads from there to the two premises forming cluster SF07/10, or in fact which premise was directly ancestral to SF11 or the spread to the farms with viruses in phylogenetic cluster SF05/13/06/16.

## Discussion

Molecular epidemiology has long been established as a useful addition to the interrogative epidemiology used to describe the movements of animals and people. There is a significant difference, however, between the global analysis of strain evolution and movements, such as has been done for influenza or SARS-CoV-2 in recent years (Petrova and Russell, 2018; COVID-19 Genomics UK (COG-UK), 2020) and the detailed analysis of a localized outbreak, such as the CSF outbreak in the UK in the year 2000. To achieve a high resolution with certainty for such localized outbreaks, a minimum number of sequence changes need to be present to obtain a phylogenic resolution. In addition, the resources available for control of animal viruses are limited compared to human viruses, such as influenza and SARS-CoV-2, especially in developing regions, thus it is important to know if efforts to gain sequence data will provide useful information.

Analysis of the full-length E2 gene, which encodes the major immunogenic glycoprotein, has been applied to study the epidemiology of CSFV and provides useful discrimination between related strains circulating over a longer period within endemic regions (Jiang et al., 2013; Postel et al., 2013). Obtaining this 1,119 nucleotide sequence by PCR and Sanger sequencing is a straightforward and efficient method to study strains isolated over a prolonged period and/or between geographical regions. However, in this study, insufficient genetic diversity was found within the E2 coding region to assist in tracing virus movements over the relatively short time frame of this outbreak. Indeed, the almost full-length genome sequences contained a maximum of 14 nucleotide changes only, indicating that as much sequence information as possible will be beneficial. Different sequences from different animals on the same premise would be indicative of the time since the incursion to the farm, but multiple incursions (e.g., through multiple infected animals) also need to be considered. To delineate true differences over a slightly longer period a farm consensus sequence might be beneficial. The data analyzed in this study indicate that significant evolutionary changes that lead to the formation of new clusters can be detected through both approaches, but further studies will be required to resolve the mutation rates of CSFV per pig against time.

The relatively high level of differences found between the sequence from a single animal on IP01 and the sequence of pooled samples from the same farm likely reflect the substantial circulation of the virus within this farm, where a diagnosis of PCV-2-associated disease was initially made, and CSF was not suspected for weeks until about one-third of the 3,600 pigs were sick and 200 had died (Paton, 2002). Similarly, we can conclude a rather early transmission of the CSFV to farm IP11, even though the origin of transmission cannot be resolved further at this moment.

Sequencing of full virus genomes is becoming faster and increasingly cost-effective and its utility for large-scale molecular epidemiology has been demonstrated for numerous viruses (Grubaugh et al., 2019b). A particularly pertinent example of tracing a single outbreak was the molecular epidemiology of the foot and mouth disease virus (FMDV) outbreaks in 2001 and 2007 in the UK, which have been studied both retrospectively and in real time (Cottam et al., 2008a,b). The real-time analysis of FMDV genomes from the 2007 outbreak identified 9 nucleotide changes in the sequence between the viruses detected in early infected premises and a later cluster of infection that occurred 5 weeks after the initial premises were culled. This number of changes was higher than the predicted mean number of changes between premises and inferred the potential for the existence of intermediate, undetected, infected premises. Field investigations subsequently identified an infected premise that was the likely link between the two clusters.

However, interpreting the significance of small numbers of nucleotide changes is difficult and there are substantial differences in the rate of accumulation of genetic mutations between viruses (Campbell et al., 2018) and, for example, FMDV has a slightly higher mutation rate than CSFV (Freimanis et al., 2016; Rios et al., 2017). Epidemiological situations also vary and may impact virus evolution. For example, the presence of wildlife virus reservoirs, other susceptible species, length of the outbreak, number of infected individuals or premises, and transmission routes will vary vastly and hence no two outbreaks, even of the same disease, will be the same. Knowledge on the extent and rate of virus evolution for different viruses under different circumstances is, therefore, important to assist in the interpretation of the significance of genetic changes.

The CSF outbreaks from various epidemiological situations have been analyzed by full genome sequencing. Leifer and colleagues sequenced isolates from wild boar in Germany in a region close to a previous vaccination zone (Leifer et al., 2010). Comparing the genome sequences of two viruses isolated in 2009 to the sequence of a putative ancestor strain, isolated 4 years earlier, inferred that, despite intense surveillance of this wildlife population, the virus had persisted undetected. Analysis of 11 genomes from viruses isolated over a 5-year period from wild boar in northern France indicated that the virus evolved relatively slowly, with an estimated substitution rate of 1.5 × 10^−3^ substitutions per site per year (Goller et al., 2016). This rate of evolution was sufficient to discriminate two spatial and temporal clusters that were separated by physical barriers and provided insight into the virus spread in wild boar over this extended time period.

More recently, full genome analysis has been applied to the CSF outbreak that started in 2018 in Japan (Sawai et al., 2021). A genotype 2.1 virus was initially detected in a farm in the Gifu prefecture and later found to be present in wild boar in the surrounding region. By 2020, the virus had spread to 65 farms and over 200 wild boar cases had been detected, with long distance transmissions occurring. Analysis of full-length genome sequences of 155 isolates, from farms and wild boar, indicated a similar virus evolution rate to earlier studies and molecular clock analysis allowed estimates of when the virus was introduced into the country, and when subsequent long distant transmission events may have occurred. Phylogeographic analysis indicated that the virus movement occurred mainly between neighboring regions, although some movements between non-adjacent areas were inferred, implying that both wild boar-mediated spread and transmission by anthropogenic factors occurred. A novel method of analysis of shared nucleotide variants allowed a greater degree of resolution between the highly similar Japanese isolates (Yamamoto et al., 2021). This method agreed well with standard phylogenetic analysis and helped define the borders between closely related groups of viruses thus enabling more detailed inferences about transmission pathways.

In summary, our study supports other recent studies that the full-length CSFV genome sequence can be useful to inform about disease control strategies, in conjunction with traditional epidemiological investigations, and that efforts to obtain and analyze such data in real time during an outbreak are warranted. Had this sequence information been available in the year 2000, it could have informed the field of epidemiological investigations substantially. However, interpretation of the significance of a small number of genetic changes remains challenging, especially if data are accumulating in real time (Villabona-Arenas et al., 2020). Greater knowledge on the rate of genetic changes occurring in the CSFV genome during different transmission events, particularly during genetic bottlenecks, such as during vertical or fomite transmission, will assist in interpreting the significance of genetic data within the epidemiological context. The potential to enhance the resolution through the analysis of intra- and inter-host single nucleotide variants will be interesting to examine in further studies that could allow to better inform on CSFV transmission pathways in real time.

## Data Availability Statement

The datasets presented in this study can be found online at: https://www.ncbi.nlm.nih.gov/, accession numbers LC702327 to LC702341.

## Author Contributions

FS and HC conceived the project and acquired funding. RS, SM, and SG contributed to sample processing, generation, and analysis of sequence data. BC performed phylogenetic analysis. HC prepared the manuscript. All authors critically reviewed the manuscript.

## Funding

The authors and these studies were financially supported by the UK Department for Environment, Food and Rural Affairs (Defra), the Scottish Government, and the Welsh Government projects SE2210 and SE2215.

## Conflict of Interest

The authors declare that the research was conducted in the absence of any commercial or financial relationships that could be construed as a potential conflict of interest.

## Publisher's Note

All claims expressed in this article are solely those of the authors and do not necessarily represent those of their affiliated organizations, or those of the publisher, the editors and the reviewers. Any product that may be evaluated in this article, or claim that may be made by its manufacturer, is not guaranteed or endorsed by the publisher.
